# Nurse health and lifestyle modification versus standard care in 40 to 70 year old regional adults: study protocol of the Management to Optimise Diabetes and mEtabolic syndrome Risk reduction via Nurse-led intervention (MODERN) randomized controlled trial

**DOI:** 10.1186/s12913-017-2769-z

**Published:** 2017-12-06

**Authors:** Melinda J. Carrington, Paul Zimmet

**Affiliations:** 1 Pre-Clinical Disease and Prevention Unit, Baker Heart and Diabetes Institute, PO Box 6492, Melbourne, Victoria 3004 Australia; 2 Centre for Primary Care and Prevention, MacKillop Institute for Health Research, Australian Catholic University, Melbourne, Victoria Australia; 3 Department of Diabetes, Faculty of Medicine, Nursing and Health Sciences, Monash University, Melbourne, Victoria Australia

**Keywords:** Metabolic syndrome, Cardio-metabolic risk factors, Cardiovascular disease, Type 2 diabetes mellitus, Nurse-led intervention, Health and lifestyle modification, Clinical trial

## Abstract

**Background:**

Metabolic syndrome (MetS), the clustering of multiple leading risk factors, predisposes individuals to increased risk for developing type 2 diabetes and/or cardiovascular disease (CVD). Cardio-metabolic disease risk increases with greater remoteness where specialist services are scarce. Nurse-led interventions are effective for the management of chronic disease. The aim of this clinical trial is to determine whether a nurse-implemented health and lifestyle modification program is more beneficial than standard care to reduce cardio-metabolic abnormalities and future risk of CVD and diabetes in individuals with MetS.

**Methods:**

MODERN is a multi-centre, open, parallel group randomized controlled trial in regional Victoria, Australia. Participants were self-selected and individuals aged 40 to 70 years with MetS who had no evidence of CVD or other chronic disease were recruited. Those attending a screening visit with any 3 or more risk factors of central obesity, dyslipidemia (high triglycerides or low high density lipoprotein cholesterol) elevated blood pressure and dysglycemia were randomized to either nurse-led health and lifestyle modification (intervention) or standard care (control). The intervention included risk factor management, health education, care planning and scheduled follow-up commensurate with level of risk. The primary cardio-metabolic end-point was achievement of risk factor thresholds to eliminate MetS or minimal clinically meaningful changes for at least 3 risk factors that characterise MetS over 2 year follow-up. Pre-specified secondary endpoints to evaluate between group variations in cardio-metabolic risk, general health and lifestyle behaviours and new onset CVD and type 2 diabetes will be evaluated. Key outcomes will be measured at baseline, 12 and 24 months via questionnaires, physical examinations, pathology and other diagnostic tests. Health economic analyses will be undertaken to establish the cost-effectiveness of the intervention.

**Discussion:**

The MODERN trial will provide evidence for the potential benefit of independent nurse-run clinics in the community and their cost-effectiveness in adults with MetS. Findings will enable more nurse-led clinics to be adopted outside of major cities and encompassing other chronic diseases as a key primary preventative initiative.

**Trial registration:**

MODERN is registered with the Australian New Zealand Clinical Trials Registry (ACTRN12616000229471) on 19 February 2016 (retrospectively registered).

**Secondary identifiers:** MODERN is an investigator-initiated trial funded by the National Health and Medical Research Council of Australia from 2014 to 2017 via a Project Grant (ID No. APP1069043) and was approved by the Australian Catholic University Human Research Ethics Committee (Project No: 2014 244 V) and the Department of Health Human Research Ethics Committee (Project No:38/2014) for the release of Medicare claims information.

## Background

Metabolic syndrome (MetS), the constellation of central obesity, dyslipidemia, elevated blood pressure (BP) and dysglycemia [1], encapsulates some of the leading non-communicable disease risk factors in the world [2]. For three of these risk factors, 9% of the Australian adult population are overweight or obese, have dyslipidemia and elevated BP in combination, with 66% of the population having three or more of any behavioural or biomedical risk factor (at the same time) for developing cardiovascular disease (CVD), diabetes and chronic kidney disease [3]. The proportion of adults with antecedent risk for cardio-metabolic disease increases with increasing remoteness [3]; this geographical pattern of inequality is reflected by higher rates of CVD and diabetes prevalence [4], deaths [5] and hospitalizations [6].

Notwithstanding the definition used to identify subjects with MetS, having multiple risk factors is detrimental to increased disease risk in a dose-response gradient with more components of the MetS [7, 8]. CVD morbidity and mortality outcomes are between 2 to 2.4-fold higher [9], and stronger for type 2 diabetes (T2DM) risk of between 3 to 5-fold greater [8] in individuals with MetS. CVD is the most common clinical sequelae in those with T2DM and MetS itself promotes the development of both of these conditions due to the association between components and atherosclerosis and dysglycemia. Fortunately, in people with MetS, a meta-analysis has demonstrated the benefits of lifestyle and pharmacological interventions for reversal of MetS [10] and decreased likelihood of developing CVD [11] and T2DM [12]. Robust evidence exists for the clinical usefulness of nurse-led interventions for improving cardio-metabolic risk factors in adults with chronic conditions [13–15], fostered by written protocols embedding clinical practice guidelines and a structured framework to enable the titration and intensification of therapy and retraction of the frequency of management when deemed appropriate [16]. However, no randomized controlled trial with standard care as the comparator over the longer term to evaluate health benefit and cost-effectiveness of community nurse-led clinics has been established to date in higher risk, non-urban settings.

Therefore, our aim was to assess the effectiveness of a nurse-led clinic program that modulated health and lifestyle factors to reduce cardio-metabolic abnormalities and future risk of CVD and T2DM in individuals with MetS. It was hypothesised that there would be more adults with MetS in the nurse-facilitated health and lifestyle modification group compared to standard care who achieve the primary endpoint of meeting target risk factor thresholds or minimum changes that would be considered clinically significant for any 3 or more risk factors that characterise the MetS over 2 year follow-up.

## Methods/Design

### Study design

**Fig. 1:**
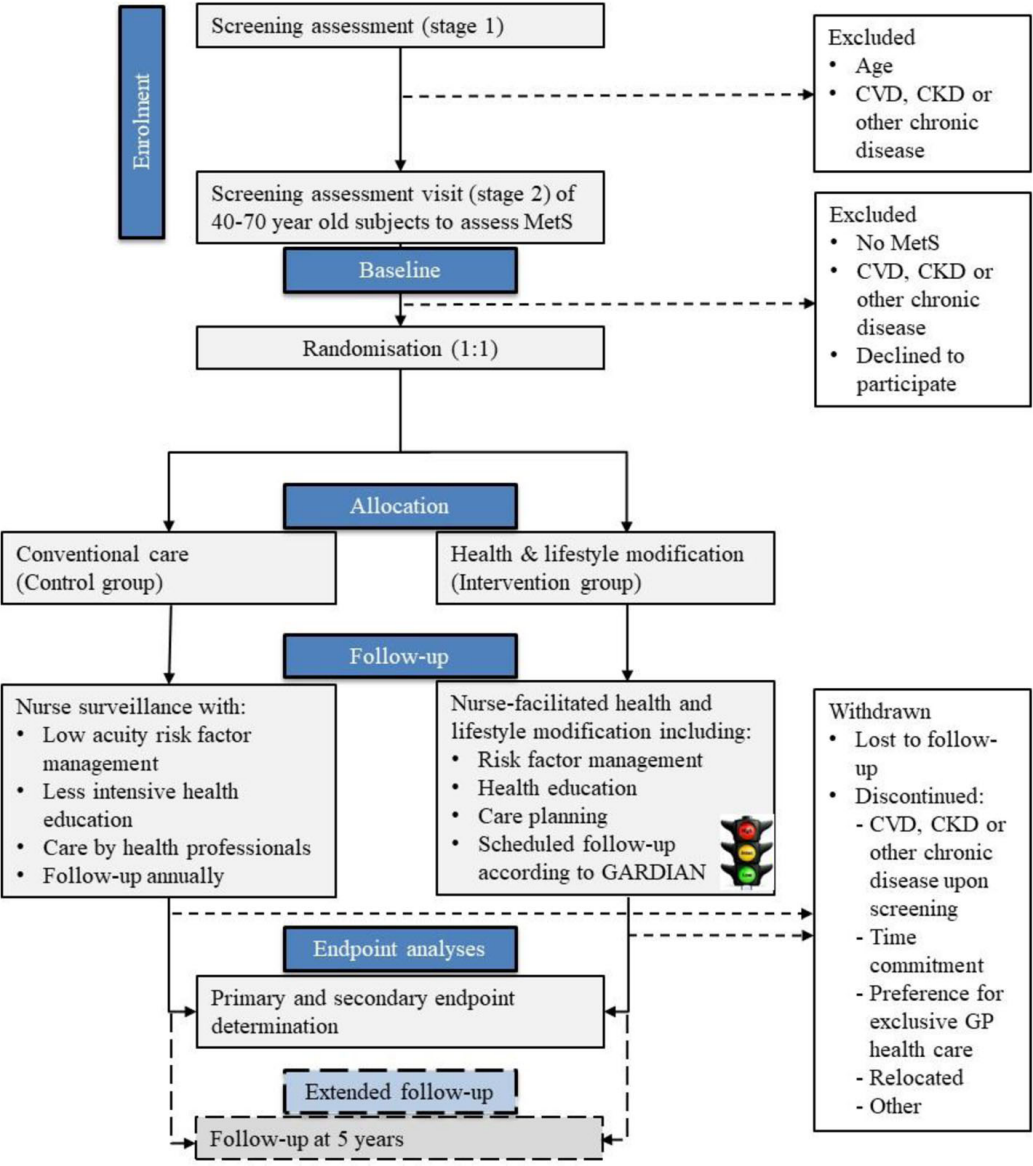
Study flow diagram from screening to endpoint evaluation after 2 years and extended follow-up to 5 years

MODERN is a multi-centre, open, parallel group randomized controlled trial evaluating the effect of nurse-facilitated health and lifestyle modification (intervention group) vs. standard care (control group) on a primary cardio-metabolic end-point (achievement of risk factor levels to eliminate MetS or minimal clinically meaningful change in risk factor levels for MetS) in at risk regional residents aged between 40 and 70 years. The study flow diagram over 2 year follow up and extended 5 year longer-term follow-up is shown in Fig. 1. The final study protocol (Version 2; 5 August 2014) was approved by the Australian Catholic University Human Research Ethics Committee (Project No: 2014 244 V). Study nurses obtained written informed consent to participate and additional consent for the release of Medicare claims information (medical services and prescriptions), as approved by the Department of Health Human Research Ethics Committee (Project No: 38/2014). Subjects could discontinue their participation upon request, in which case personal information collected up to the time of withdrawal would be retained for analyses, unless the participant stated otherwise.

### Study population and recruitment strategy

Participants were self-selected from two regional locations in Shepparton, northern Victoria, and Colac, south-west Victoria (estimated resident population 40–70 years at 30 June 2015 was 23,437 and 13,055, respectively). Census data were used to ensure that a representative sample of the age and sex distribution of each regional community between 40 and 70 years were recruited. The sampling strategy involved large scale promotion of the study to key local workplaces, community groups (e.g. Rotary) and Local Members of Parliament, community engagement at local markets and stalls, direct and indirect postal invitations, radio and newspaper advertising and social media (e.g. Facebook). Individuals responded to participate and a brief screening questionnaire administered via telephone or face-to-face was initially used to determine age and history of CVD or other chronic disease. Eligible participants made an appointment to undergo screening to identify MetS from 26 September 2014 and recruitment was completed on 1 April 2016. The study protocol conformed to SPIRIT guidelines and a full CONSORT diagram will be finalised upon trial completion.

### Selection criteria

Participants residing in, or in surrounding regions of Shepparton or Colac and aged between 40 and 70 years with any three or more risk factors for MetS [1] as shown in Table 1 at the time of screening were eligible. The equivalent HbA_1C_ level for increased risk of diabetes was used as a substitute for fasting plasma glucose [17]. Participants were included if they were capable of attending scheduled study clinic visits and able to provide informed consent. Participants outside the eligible age range with two or less identifiable risk factors, any diagnosed form of CVD, chronic kidney disease or other forms of chronic disease that resulted in the belief that participation would not be appropriate, were excluded. Participants were omitted from participating if they had neurological/cognitive impairment and/or were unable to provide written informed consent.

**Table 1:** Criteria for metabolic syndrome classification [1]

Risk factor for MetS	Cut-point criteria
Elevated WC	Men: > 94 cm
	Women: > 80 cm
Elevated triglycerides	≥ 1.7 mmol/L
Reduced HDL-C	Men: < 1.03 mmol/L
	Women: < 1.29 mmol/L
Elevated BP	≥ 130/85 mmHg
Elevated HbA_1C_	≥ 5.7% (39 mmol/L)

*MetS* metabolic syndrome, *WC* waist circumference, *HDL-C* high-density lipoprotein cholesterol, *BP* blood pressure, *HbA*_*1C*_ glycated haemoglobin

### Randomisation

Participants who satisfied the selection criteria were randomized into one of two trial arms according to a 1:1 allocation ratio using a pre-determined computer generated sequentially numbered randomisation schedule centrally performed via SPSS Statistics 22.0 (SPSS Inc., IL, USA) and transferred to the receptionist at each study clinic to assign group distribution. Group allocation was concealed from nurses to eliminate selection bias during recruitment. Block randomisation (per regional study clinic) occurred with block sizes of 20 and stratified according to MetS without T2DM or with a diagnosis of T2DM. Due to the nature of the intervention, blinding subjects and nurses was not possible.

### Primary endpoint

The primary endpoint was the between group difference in achievement of the target risk factor thresholds (Table 1) or minimum changes shown in Table 2 that would be considered clinically significant for any 3 or more of the risk factors that characterise the MetS, These clinically significant minimum changes represent > 0.5 standard deviation change from baseline, calculated utilising pilot data from adults with MetS [18].

**Table 2:** Criteria for minimum change in components of metabolic syndrome to assess the primary endpoint

Risk factor for MetS	Clinically significant minimum change	
	Men	Women
Elevated WC	Reduce by ≥ 5 cm	Reduce by ≥ 6 cm
Elevated triglycerides	Reduce by ≥ 0.6 mmol/L	Reduce by ≥ 0.5 mmol/L
Reduced HDL-C	Increase by ≥ 0.15 mmol/L	Increase by ≥ 0.18 mmol/L
Elevated BP	Reduce by ≥ 7/3 mmHg	Reduce by ≥ 8/4 mmHg
Elevated HbA_1C_	Reduce by ≥ 0.4%	Reduce by ≥ 0.5%

*MetS* metabolic syndrome, *WC* waist circumference, *HDL-C* high-density lipoprotein cholesterol, *BP* blood pressure, *HbA*_*1C*_ glycated haemoglobin

### Secondary endpoints

Secondary endpoints included between group changes at 24 months in meeting the individual components of the primary endpoint (i.e. target thresholds and clinically significant changes); resolution of MetS status; incidence of T2DM, as indicated by initiation of glucose-lowering medication following confirmation of a diagnosis of T2DM or a HbA_1C_ level ≥ 6.5% [17]; incidence of CVD, defined by fatal events, a diagnosis of CVD or non-fatal CVD-related events requiring hospitalization; within and between group variations in health behaviours, general health and cardio-metabolic risk factors, and; health economic analyses of the utilisation and cost of pharmacological therapies, community health care, and cardio-metabolic specific medical interventions and hospitalizations to establish the cost-effectiveness of the MODERN intervention.

### Procedures

A self-report questionnaire posted to participants in the week prior to their scheduled visit collected information regarding: *socio-demographic factors* including marital and work status, primary language spoken, income, education and ethnicity; *health behaviours* including smoking, diet and alcohol via the Dietary Questionnaire for Epidemiological Studies (DQES v2) [19], sleep and physical activity and sedentary behaviour via the International Physical Activity Questionnaire (IPAQ) [20] and; *general health* including history of CVD, associated conditions or other serious conditions, family history of CVD or diabetes, prescribed medications and adherence via the Medication Adherence Questionnaire (MAQ) [21], signs and symptoms of angina via the Rose Angina questionnaire [22], health related quality of life via the Assessment of Quality of Life – 8 Dimension (AQoL-8D) [23], perceived risk and health belief evaluation via item specific self-efficacy [24] and locus of control [25].

An on-line, discrete choice experiment during the week prior to their scheduled visit at baseline and at study end evaluated participant *preferences* for the attributes of a health and lifestyle management program they consider of greatest importance affecting their choice to participate in such programs.

The *screening assessments* were undertaken at a dedicated clinic or on-site at workplaces (for few organisations with large numbers of workers) by registered nurses according to a standardised protocol at baseline, prior to randomisation. The same registered nurse who conducted the screening assessment remained responsible for the follow-up assessments of each participant until study completion; due to standardising the management of participants and frequency of follow-up, a different nurse that managed participants from only one study group was not required to alleviate bias.

Waist and hip circumference were measured using a Figure Finder® Tape Measure (Novel Products, IL, USA) in accord with the World Health Organization (WHO) STEPwise approach to surveillance (STEPS) procedure [26] in the horizontal plane whilst standing; the level mid-way between the lowest rib and iliac crest at the end of a gentle expiration was taken for waist circumference and the level at the maximum extension of the buttocks defined hip circumference. Body mass index (BMI, kg/m^2^) was determined from height, measured using a portable stadiometer (seca®, Hamburg, Germany) and weight, using digital weighing scales (A&D Medical, SA, Aus), after the removal of shoes and heavy garments. Total cholesterol (TC), high- (HDL-C) and low- (LDL-C) density lipoprotein cholesterol, triglycerides and HbA_1C_ in capillary/fingerstick whole blood were analysed by the reflectance photometry technique using a calibrated Afinion™ AS100 analyser (Alere, MA, USA). Sitting blood pressure (BP) was measured in the brachial artery with a suitable cuff size using a calibrated Omron HEM-907 automated monitor (Omron Healthcare Co. Ltd., Kyoto, Japan) after 5 min of rest; the average of two measurements separated by a one-minute interval were analysed provided BP did not vary by ≥ 10/≥ 5 mmHg, in which case another reading was taken and the closest two readings were analysed. Calculation of CVD risk via the Framingham Risk Equation [27], and T2DM risk via the AUSDRISK tool [28], was estimated from applicable health and lifestyle risk factors and results from the thorough screening assessment to predict risk of a cardiovascular event [29] and developing T2DM [28], respectively, over the next 5 years.

If MetS was indicated and subjects agreed to be randomized into a study group, additional information was collected including: *electrocardiography* (ECG) via a 12-lead Universal ECG™ in accordance with standard electrode placement and recorded using Office Medic™ Software (QRS Diagnostic, MN, USA); *biochemistry* assessment from venous blood of creatinine to estimate glomerular filtration rate (eGFR) using the Modification of Diet in Renal Disease study equation, and high-sensitive C-reactive protein (hs-CRP) as an inflammatory marker of CVD risk; *actigraphy* for 7-day continuous quantitative measurement of sleep, physical activity and sedentary behaviour using GTX3 accelerometers (ActiGraph, FL, USA) and; *arterial stiffness* from ankle brachial (ABI) and cardio ankle vascular pressure indexes (CAVI) using the VaSera™ VS-1500 N vascular device (Fukuda Denshi Co Ltd., Tokyo, Japan).

Qualitative evaluation of intervention fidelity [30] to assess 1) adherence to key aspects of the protocol being implemented, and 2) competence in delivering the program in terms of communication, technical abilities and skills in responding to participants was established by video recordings of nurse/participant clinic visit interactions that were later evaluated by two trained observers, with feedback of results to improve nurse’s performance. For selection of a random set of observations to provide a representative sample for measuring intervention fidelity, nurses were advised (3–5 days prior) by the study co-ordinator that all participants scheduled to attend clinic visits in the following week will be asked to provide written informed consent to video recording their clinic encounter. Fidelity monitoring continued until the target number of 2 observations from each of the two trial arms (4 in total) per nurse at baseline and mid (12 month) study visits was completed.

### Intervention group – Health and lifestyle modification

**Fig. 2:**
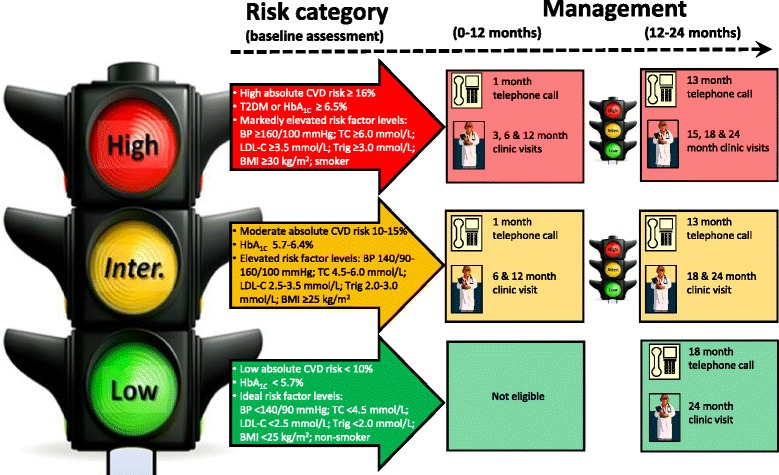
GARDIAN-guided classification of risk and follow-up plan over 24 months

The nurse-facilitated intervention required that two nurses at each site actively deliver a health and lifestyle intervention and remain responsible for assessing participant progress in achieving their health goals until study completion. Nurses applied the intervention on an individual basis and acted within the scope of their nursing responsibilities and independent of the participant’s health care professionals. The aim of the intervention was to deliver coaching to support individuals to achieve health behaviour change for improved risk factor management. Intervention participants were permitted to seek any relevant concomitant care. Key components were:

1. Cardio-metabolic risk factor management - to achieve ideal goal levels based on recommended guidelines and with consideration for the circumstances of unique individuals. For weight management, waist circumference, BMI measurements and responses to the DQES food frequency questionnaire were all evaluated followed by tailored advice on healthy eating in accord with Australian Dietary Guidelines [31] to strive for a healthy weight range of BMI 18.5–24.9 kg/m^2^ or waist circumference < 80 cm (women) or < 94 cm (men). For physical activity, responses to the IPAQ were assessed and according to Australian Government Department of Health guidelines [32], subjects were advised to aim towards accumulating at least 30 min of moderate intensity physical activity on all or most days of the week and if capable, vigorous activity for 30 min or more for 3 or 4 days a week, and to limit sedentary behaviour. For dyslipidaemia (defined as not meeting any one target lipid level) and dysglycemia, results from fasting point of care profiling were reviewed to achieve optimal levels for reduced risk of coronary artery disease [33] of < 4.5 mmol/L for TC, < 2.5 mmol/L for LDL-C, > 1.0 mmol/L for HDL-C, and < 2.0 mmol/L for triglycerides, and for reduced risk of diabetes [17] of < 5.7% (39 mmol/mol) for HbA_1C_. For BP, average clinic readings were classified in accord with National Heart Foundation guidelines [34] with the aim to achieve a BP of < 140/90 mmHg. Smokers were assessed on their readiness to cease smoking and provided appropriate counselling to encourage them to attempt to quit. People who chose to drink alcohol were advised to limit their intake to no more than 2 standard drinks on any day to reduce the lifetime risk of alcohol-related harm in accord with Australian Guidelines for Alcohol Consumption [35]. For overall CVD and T2DM health, absolute CVD and T2DM calculated risk levels were categorised in accord with recommended guidelines [28, 29] with the goal to reduce risk of CVD and T2DM. Diet and lifestyle modification was initially indicated for all subjects with dyslipidemia, dysglycemia and elevated BP, regardless of CVD and T2DM risk, except in the case of extreme results whereby participants were referred to their nominated primary health practitioner for consideration for drug therapy or additional clinical care.
  The results of the baseline screening assessment were assessed in accord with the Green, Amber, Red Delineation of Risk and Need (GARDIAN) management system to determine the frequency of health care intervention over study follow up [16]. Figure 2 shows that eligible intervention participants were designated a traffic light colour RED (high) or AMBER (intermediate) [noting the unavailability of GREEN (low) at baseline since eligibility required the presence of risk factors] to denote their overall degree of risk and corresponding GARDIAN-guided level of nurse intervention. GARDIAN designated RED subjects as those with high absolute CVD risk scores ≥16%, T2DM or identification of diabetes by HbA_1C_ ≥ 6.5%, markedly elevated levels of individual risk factors or who were smokers. GARDIAN designated AMBER subjects were those with moderate absolute CVD risk scores 10–15% or with risk factors above recommended ranges. GARDIAN designated GREEN subjects (at 12 month review) were those identified by low absolute CVD risk scores < 10%, HbA_1C_ < 5.7% or risk factor levels within recommended limits.
2. Health education – to put risk factor management in context, participants received education on CVD and T2DM including causes, risks, symptoms, treatment and management; interpretation of individualised test results in contrast to target goal levels and benefits of changing behaviour(s); understanding how to read food labels; managing portion serving sizes and comprehension and adherence to prescribed medication. All participants received a brief report containing their risk assessment results as well as a Health Passport to document before and after photographs, record appointment times and to maintain a “health identity” profile to track risk factor measurements over time and change to their GARDIAN (traffic light) risk colour code. Written education information and risk factor targets to aim for to remain healthy was consolidated within the Health Passport.
3. Care planning – to help reduce identified risk factors, nurses trained in motivational interviewing [36] applied the 5As model (assess, advise, agree, assist, arrange) [37] of behavioural counselling and prompted participants to develop self-care and management plans to stimulate behaviour change. With nurse support, participants’ motivations to change, including self-nominated goals/priority areas and obstacles or catalysts to achieve them were identified. Attainable targets to achieve individualised health goals were agreed and suitable community lifestyle programs or health professional referral for improved care were recommended, with formal re-evaluation of care plans mid-study.
4. Scheduled follow-up - to assist subjects to adhere to the care plan and reinforce positive behaviour change, specified follow-up visits and supplemental telephone coaching was implemented in accord with GARDIAN status. The frequency of contact with participants was aligned with their level of risk. As shown in Fig. 2, GARDIAN designated RED subjects at baseline received a telephone call at 1 month and clinic visits at 3, 6 and 12 months. GARDIAN designated AMBER subjects received a telephone call at 1 month and clinic visits at 6 and 12 months. After 12 months, screening assessments were repeated and GARDIAN classification was reassessed; revised RED-coded subjects received a telephone call at 13 months and clinic visits at 15, 18 and 24 months. Updated AMBER coded subjects received a telephone call at 13 months and clinic visits at 18 and 24 months. Newly designated GREEN subjects received a telephone call at 18 months and a clinic visit at 24 months.

### Control group - standard care

Participants allocated to the standard care arm of MODERN received screening assessments but diluted risk factor management which was limited to receiving a brief risk assessment report and Health Passport with less intensive CVD and T2DM education and dietary and lifestyle advice. Ideal risk factor targets were identical yet care plans were not developed. GARDIAN status was not used to direct management and follow-up contacts at set times; aside from baseline, 12 month (mid), and 24 month (end) study visits, all management and follow-up was at the discretion of their usual health care providers. As per intervention group participants, control group participants were permitted to seek any relevant concomitant care.

### Follow-up visit format

For all participants randomized into a study group, self-report questionnaires posted 1 week prior to their scheduled visit collected updated information regarding health behaviours and general health at 12 and 24 month visits. Screening assessments, biochemistry, actigraphy and arterial stiffness tests were also repeated at 12 and 24 month visits for all participants. For intervention participants only, just screening assessments were conducted at all other (GARDIAN directed) clinic visits to help assess participant’s progress in achieving their health goals. Extended surveillance to 5 years post randomization of cardio-metabolic health and associated health care activity to determine the longer-term impact of the MODERN intervention is planned.

### Study power

A 15% difference (20% intervention, 5% control) in the number of subjects who achieved target risk factor thresholds or clinically significant changes in any 3 MetS risk factors was derived from sub-group analyses (of individuals identified with MetS) from a pilot intervention study [18] and a randomized controlled trial of a similar lifestyle intervention delivered by health professionals versus standard care [12]. With two-sided α = 0.05, an estimated minimum of 125 participants per study arm would provide 95% power to detect a 0.15 difference in the primary endpoint after 2 year follow-up. Accounting for potential loss-to-follow up of 15%, overall the trial would require approximately 150 participants randomized in each group.

### Data management, confidentiality and dissemination

Information collected under unique study identification will be returned to an independent data management unit for double data entry, range checks for data values, data queries to be resolved by data lock and coding according to standard operating procedures. Storage of paper documents will be filed in a locked compactus controlled by proxy access to authorised personnel. Electronic files will be saved in a secure server storage system that is accessible by authorised personnel in accord with information technology security processes. Data sharing and use by organisations involved in the study is guaranteed by clinical trial research agreements between collaborating institutions. Upon study completion, participants will receive a summary of group results and study findings will be presented in publications and presentations.

### Statistical analyses

All analyses will be performed according to a statistical analysis plan and on an intention-to-treat basis. Tests will be two-sided at the nominal level α = 0.05. Data from each clinic will be pooled and summarised. Descriptive statistics will summarise the socio-demographic, clinical and overall health profile of the sample at baseline and 24 months follow-up; discrete variables will be assessed by frequencies and percentages and group differences tested using χ^2^ tests with calculation of odds ratios and 95% CI, whereas continuous variables will be assessed using mean (SD) or median (IQR) and group differences compared using independent t-tests or Mann-Whitney tests for variables with skewed distributions. For dichotomous variables, multiple logistic regression using 24 month results as dependent variables will evaluate the independent correlates of achieving the primary and secondary endpoints, after adjusting for baseline results and socio-demographic characteristics. For continuous variables, mixed-model ANOVA will be performed to asses within (pre-post) and between-group (control-intervention) differences over time and multiple linear regression used to evaluate important predictors of response.

## Discussion

This study will be the first Australian-based trial to establish the evidence for the potential benefit of independent nurse-run clinics in the community and their cost-effectiveness in adults with common cardio-metabolic abnormalities. Findings will determine whether a nurse-implemented health and lifestyle modification program is more effective at improving MetS components and other major risk factors for the development of CVD and T2DM compared to standard care in adults who are in jeopardy the most by living away from urban areas where socioeconomic disadvantage is higher and specialist health care increasingly scarce [38, 39]. Importantly, our longer term study over 2-years with re-evaluation at 5 years of changes in health outcomes and health care utilisation will assess the enduring effect of nurse education and self-care and management coaching on CVD and T2DM prevention. Pending positive results and cost-effectiveness, we envisage more nurse-led clinics to be adopted outside of major cities and encompassing other chronic diseases as a key primary preventative initiative.

The novelty of the MODERN study will establish the feasibility of nurses to safely and effectively screen for the presence of MetS and deliver cardio-metabolic management based on a written protocol that integrates a systematic approach to individual assessment of risk and tailored management [16]. Nurse-led models of care provide the opportunity to maximise the use of finite resources and reserve health professional contacts for higher risk individuals. The response to the MODERN trial results however should not discount the role of general practitioners and specialists in managing chronic disease. In practical terms, MODERN will provide a model that improves the earlier detection and quality of preventative care through clear therapeutic targets for lifestyle change and risk factors, techniques to instigate behaviour change, schedules of follow-up visits and guidance to seek additional GP (or other health professional) input. With MetS driving the twin global epidemics of CVD and T2DM and the extent of the underlying burden imposed by MetS, the model being tested represents a pragmatic utilisation of the potential for nurses to manage cardio-metabolic disease and prevent or delay the development of CVD and/or T2DM.

The detailed health economic evaluation component of the MODERN trial has the distinct advantage to enhance the translation of research findings into sustainable improvements in routine clinical practice and patient outcomes. Supporting health economic analyses are lacking in the Australian context and evidence relating to the cost-effectiveness of nurse-led clinics remains an obstacle to overcome before next generation models of care can improve the quality of preventative health. At present, it is incontrovertible that components of the MetS cause risk for CVD and T2DM and the MetS is an ideal target for risk reduction programs, carried out by nurses with the competence and skill set that uphold cardio-metabolic disease prevention. The results of the MODERN trial will add to the evidence-base for the success or failure of a nurse-led health service delivery of care for combatting the likelihood of CVD and T2DM in individuals with the MetS.
